# The effectiveness of a rational-emotive intervention on teachers’ unconditional self-acceptance, perfectionism, and pupil control ideology

**DOI:** 10.3389/fpsyg.2023.1240269

**Published:** 2023-12-13

**Authors:** Elena Mirela Samfira, Florin Alin Sava

**Affiliations:** ^1^Teacher Training Department, University of Life Sciences from Timisoara, Timisoara, Romania; ^2^Department of Psychology, West University of Timisoara, Timisoara, Romania

**Keywords:** teachers, unconditional self-acceptance, perfectionism, pupil control ideology, rational emotive behavioral therapy

## Abstract

**Introduction:**

The present research aimed to investigate the effectiveness of a Rational Emotive Behavior Therapy (REBT) intervention on in-service teachers.

**Methods:**

A quasi-experimental 2-group (intervention vs. control) × 3-time (pre, post-test, follow-up) design was applied to explore to what extent the REBT interventions help teachers increase their level of unconditional self-acceptance as the primary outcome and decrease their perfectionism tendencies and pupil control ideology as secondary outcomes. The sample consisted of 100 in-service teachers assigned to either the intervention group (*n* = 50) or the control group (*n* = 50). The experimental group received a 6-week intervention program. Every session was held weekly and lasted 90–120 min. The Unconditional Self-Acceptance Questionnaire (USAQ), Pupil Control Ideology Scale (PCI), and Perfectionism Inventory Scale (PI) were used to collect data. This study used a mixed model ANOVA 2 × 3 for data analysis.

**Results:**

The results indicated that in the experimental group, there was a statistically significant increase in unconditional self-acceptance level from pre-test to post-test, which remains significant at the 6-month follow-up. Likewise, there were no statistically significant differences in unconditional self-acceptance levels between the post-test and 6-month follow-up in the intervention group.

**Discussion:**

These findings prove that REBT interventions are effective in increasing teachers’ unconditional self-acceptance.

## 1 Introduction

In the context of globally fast changes, including the educational context, the teaching profession is considered to be a very stressful profession (Brady and Wilson, 2022), among the high-risk jobs for mental disorders (Jerrim et al., 2020), stress being considered as a key factor in leaving the profession (Brady and Wilson, 2022). Teachers’ stress has a lot of negative consequences: job turnover (Kim et al., 2020), job performance and school effectiveness (Asaloei et al., 2020), anxiety (Potter, 2021), and burnout (Candeias et al., 2021). Teachers have to face the demands coming from (1) *the school principal*, who wants very good school results from the students, for a higher place in the school hierarchy; (2) *parents*, who want their children to have the most effective teaching methods, to gain a high level of information required for excellent school results; (3) *students*, who want well-trained teachers, applying technology in teaching, knowing how to manage classroom conflicts and other problematic situations; and (4) *society*, who wants from a teacher to be a good educator, a good parent, a good husband/wife, a model citizen because she/he is qualified to educate. Besides the fact that it involves so many responsibilities and a high level of stress, being a teacher is a profession with a fairly low level of rewards, compared to other professions that involve an equal number of years of study (Toropova et al., 2021).

Every teacher perceives these stress sources differently depending on the teacher’s personality, beliefs, and ability to cope with stressful situations. Also, teachers’ stress was identified as a strong predictor of teachers’ PCI (Aftab and Khatoon, 2013), and teachers’ authoritarian attitude (punishment as a strategy to disciple students) was identified as being positively related to high levels of stress and frustration (Bernard, 2016). Furthermore, in their study, Samfira and Sava (2021), concluded that teachers’ authoritarian/custodial ideology is also positively correlated with irrational beliefs, insufficient self-control, hypercriticism, and perfectionism (high standards for others, approval seeking, concern over mistakes, and perfectionistic automatic thoughts) and negatively correlated with unconditional self-acceptance.

Because of the high relevance of stress in the teaching profession, many studies have focused on interventions meant to reduce stress (see Obiweluozo et al., 2021 for REBT-based interventions; Bonde et al., 2022 for mindfulness-based interventions; Dike et al., 2021 for cognitive behavioral therapy and yoga interventions) or increase the well-being of teachers (Kidger et al., 2021). Whereas mindfulness meditation promotes self-compassion, it is not the only way to promote self-compassion. Albert Ellis, the founder of rational emotional behavioral therapy (REBT) (Ellis, 1977), highlighted the role of unconditional self-acceptance, a closely related term to self-compassion, as being a healthy concept, a rational response to irrational thoughts related to self-evaluation and self-esteem.

The current study is the first attempt to investigate the effectiveness of an REBT program aiming at increasing the level of unconditional self-acceptance in teachers, as this construct is essential for good mental health both based on theoretical accounts (Ellis, 1994) and empirical pieces of evidence as provided subsequently. Likewise, an intervention that primarily addresses the aim of increasing unconditional self-acceptance could also provide indirect positive effects on other constructs, such as teachers’ level of perfectionism and the teachers’ pupil control ideology (PCI), such constructs being relevant to teachers’ well-being and positive teacher-student interaction.

### 1.1 Teachers’ irrational beliefs

Irrational beliefs represent a very important concept in psychology literature. Irrational beliefs are considered rigid, extreme, and illogical (Evans et al., 2018) and represent non-preferential dogmatic evaluations of adverse situations, being at the same time considered unrealistic and powerful cognitions that lead to self-destructive behaviors and emotions (Trip et al., 2021). It is recognized that in many situations cultural stereotypes contribute to the development of irrational thinking (Beeghly, 2021). Ellis (2019a), analyzing irrational and rational beliefs, sustained that an individual’s irrational beliefs have a high biological basis than his rational ones and that irrational beliefs are more involved in individuals’ mental health problems.

The teaching profession, by its nature, generates specific irrational beliefs. Studies that addressed teachers’ irrational beliefs are very numerous (Nwabuko et al., 2020; Samfira and Sava, 2021). Authors in this research area concluded that teachers’ irrational beliefs are strongly related to well-being (Ifelunni et al., 2022) and burnout or negative emotions, which impede the teacher’s performance and affect the relationships with students (Huk et al., 2019). Also, Samfira and Sava (2021) found that teachers who manifest a high level of irrational beliefs have a custodial view of pupil control ideology that can be seen as profession-specific control beliefs.

Bernard (2016) identified a plethora of teachers’ irrational beliefs such as: I must have constant approval from students, other teachers, administrators, and parents; events in my classroom should always go exactly the way I want them to; People who misbehave deserve severe punishment; those who do not do well at school are worthless; I must be in total control of my class at all times; I must find the perfect solution to all problems; I must be a perfect teacher and never make mistakes (see p. 211). Unfortunately, we still find them nowadays (Schellings et al., 2023).

Teachers’ expectations that students “must” behave well, generally lead to frustration and failure. The presence of “must” represents a classic example of the use of irrational beliefs (Ellis, 2019b). Wilde (1996, p. 138–139) identified that teachers’ irrationality is characterized by self-downing, demandingness, and catastrophizing, sustaining that numerous teachers believe that to be a good teacher means to be able to control the classroom and that their value as an individual is related to their success as a teacher: I have to be perfect all the time; If I fail as a teacher, I fail in life; Pupils who are behaving improperly are bad, or Pupils who are behaving improperly are bad. Afterward, Bernard (2016) identified four categories of irrational beliefs in teachers: (1) Self-downing; (2) authoritarianism; (3) demands for justice; (4) low frustration tolerance. The self-downing sub-scale refers to the very high stands that different teachers have set for themselves, which leads to the need for social approval. The authoritarianism subscale is related to teachers’ intransigence towards their pupils’ misbehaviors. Authoritarian teachers sustain severe punishment because they are not able to cope with students’ misbehaviors. The demand for justice subscale refers to the teachers’ needs to be listened to, to have an ideal collaboration with administrators, and to be involved in decision-making procedures. The last subscale low frustration tolerance is related to teachers’ assumptions about teaching struggles. Many teachers tend to view teaching as a complicated and complex procedure, which requires a lot of effort on their part (Gkontelos et al., 2021). All these irrational beliefs are generally manifested as a reaction to four domains of teaching: classroom discipline, student learning, time and work pressure, and school administration-related issues (Bernard, 2016).

### 1.2 Teachers’ unconditional self-acceptance

Different cognitive-behavioral interventions have been applied to identify and change irrational beliefs that hinder teachers’ performance in the classroom (Ifelunni et al., 2022). Chadha et al. (2019) sustain that Rational Emotive Behavior Therapy (REBT), developed by Ellis (1957), shows that an individual’s appraisals of specific situations generate different emotional reactions and that individuals’ psychological disturbance is determined by using different irrational thoughts processes in developing those appraisals. REBT is effective in the educational context because it helps teachers learn to control their emotions, reduce dysfunctional distress and job burnout, and behave in an acceptable manner in response to students’ challenging behaviors (Onuigbo et al., 2020). The role of REBT in education is highlighted even by Ellis who stated that “I have always believed in the potential of REBT to be used in schools as a form of mental health promotion” (Ellis and Bernard, 2006, p. ix).

Ellis (1994) proposed a healthy concept, a rational response to irrational thoughts, related to self-evaluation and self-esteem defined as unconditional self-acceptance, meaning that “the individual fully and unconditionally accepts himself whether or not he behaves intelligently, correctly, or competently and whether or not other people approve, respect, or love him” (p. 101). Translated into beliefs, self-acceptance represents the rational belief that an individual is a valuable person just for existing, despite all the flaws and mistakes she/he does, this construct being considered essential for mental health (Ellis, 2019a) because individuals are satisfied with themselves.

What is necessary to highlight about the concept of unconditional self-acceptance is that being subject to mistakes, no individual works and behaves perfectly (Ellis, 2019a), and this aspect could prevent or reduce perfectionism. If an individual fails to perform very well in her/his work, does not make him automatically an unsuccessful person (Ellis, 2019a). In this case, the individual should consider that her/his performance was low, not that she/he is an unsuccessful individual (Artiran, 2019). But, at the same time, it is important to clarify that this type of mentality does not automatically lead to resignation, which assumes that an individual’s weaknesses are appreciated or ignored (Dryden and Neenan, 2020) but rather involves active involvement in the struggle to diminish and/or eliminate weaknesses (Ellis, 2019a).

Research on unconditional self-acceptance found that a low level of unconditional self-acceptance correlates with depression, anxiety, anger, and neuroticism (Popov, 2019; Prihadi et al., 2019; Andronikos, 2021). High levels of unconditional self-acceptance were in general strongly related to mental health, happiness, self-esteem, life satisfaction, well-being, respect for the differences between individuals, and low levels of anxiety and depression (Vural-Batik, 2019; Bernard M. E., 2020). Unconditional self-acceptance also correlates positively with dispositional forgiveness (Porada et al., 2018), self-compassion and flourishing (Venet, 2019; Andronikos, 2021). These results could be used in counseling teachers when they show certain uncontrolled reactions towards some students, which they cannot explain later in discussions with the principals or parents.

In the educational context, different researchers showed that teachers’ self-acceptance was positively correlated with well-being and mindfulness and negatively correlated with perceived stress and burnout (Bingöl and Batik, 2019; Sun et al., 2019; Corcoran and O'Flaherty, 2022). It is auspicious to mention that the decision to unconditionally accept ourselves can be viewed like any other decision we make in our life. At any moment, non-self-accepting individuals can decide to accept themselves as they are, all they need is to be willing to see their own life from multiple perspectives, and then, change those perspectives (Gran, 2021). Unconditional self-acceptance incorporates the acceptance of oneself as a whole, without concern about approvals from other individuals (Bingöl and Batik, 2019). An unconditional teacher is not afraid to be authentic in relations with their students, to be a humanistic person (not only a humanistic teacher) rather than a controlling/authoritarian figure (Samfira and Sava, 2021). Likewise, as self-acceptance is related to the acceptance of others (Porada et al., 2018). Akaki et al. (2020) concluded that acceptance of others follows self-acceptance, a principle that should be taken into consideration when we have the intention to help individuals to accept others (e.g., at home or at work) for who they are. An individual who accepts others does not judge them, because nobody is perfect, does not try to change others, because every person has his own ideas, and avoids resentment, especially when they are in a superior position. The benefits of accepting others “as they are” could build strong relationships with students, could facilitate the understanding of another point of view, and could reduce the need of controling others (Lapshin, 2020).

One of the most important aspects of educational context is that increasing the level of unconditional self-acceptance has a positive effect not only on teachers but even on their students. In the same vein, Mitchell et al. (2018) sustained that pupils who felt that there are unconditionally accepted by their teachers were more inclined to be interested in the learning process and to develop an emotional attachment to school. One explanation is given by Venet (2019) who highlights that unconditional acceptance represents what children require to flourish. Additionally, the voice of teachers from different research (Kohn, 2005) concluded that unconditional acceptance represents an effective approach to helping pupils to become better individuals. Pre-service teachers and in-service teachers must be learned to make the difference between accepting pupils for *who* they are, not for *what* they do. In doing so, teachers will respond not only to students’ different needs (emotional, social, or physical) but to a *whole* child, to an integrated self (Kohn, 2005). An optimistic aspect for teachers is to know that every stage of life could represent an opportunity to develop a higher level of unconditional self-acceptance (Guterman, 2020).

All these principles can be transferred into an educational context, where the level of teachers’ unconditional acceptance could be seen as a valuable preventive resource in the successful teacher-student relationship, which has not been of much interest to researchers. An unconditional teacher is not afraid to behave with their students as she/he really is, being a more humanistic person than a controlling/authoritarian person (Samfira and Sava, 2021). Understanding the importance of unconditional self-acceptance in education, Kohn (2005) developed the concept of “unconditional teaching.” In this regard, the author sustained that many teachers, because they are human beings, may not like all their students, but the difference for an unconditional teacher is that she/he tries hard not to play favorites (Kohn, 2005). Developing positive relationships with students requires not only that teachers unconditionally accept themselves, but also unconditionally accept their students. Even for students is also important to feel unconditionally accepted, by their teachers, because they are more likely to be involved in their learning activities and to develop an emotional attachment to school (Mitchell et al., 2018). Not only students but also pre-service teachers have the same expectations that their university teachers to unconditionally accept them and try to help them in their preparation for the teaching profession (Sevim et al., 2020).

Sezer et al. (2020) in their research conducted with school principals, found that unconditional acceptance, as a professional value, should be gained and developed through in-service training, to help teachers to understand the positive and negative consequences of this concept, both on themselves and on their students. Despite the high relevance for unconditional acceptance in educational settings there is a lack of studies on interventions aimed to increase the unconditional acceptance applied to teachers. Only articles applied to children, adolescents (Bernard et al., 2013), and students (Godin, 2010) has been identified so far.

The negative correlation between teachers’ unconditional self-acceptance and irrational beliefs, anxiety, stress, depression, burnout, and perfectionism (Samfira and Sava, 2021; Yeo, 2022) and positive relationship with teachers’ well-being (Bingöl and Batik, 2019), helped us considering that an intervention for increasing the unconditional self-acceptance is needed and appropriated for several important issue in the teaching profession.

### 1.3 Teachers’ perfectionism

Perfectionism, as a complex and multidimensional personality trait, has received increased attention in the last decades, being widely investigated within different contexts (Hill et al., 2016; Stoeber, 2017; Samfira and Paloş, 2021). Experts define perfectionism as representing an unrealistically and exceedingly high standard of performance, if not impossible to meet, overly critical self-evaluation, over-sensitivity about others’ evaluations, and a focus and striving for flawlessness (Stoeber et al., 2021). In a cross-temporal meta-analysis (1989–2016), conducted by Curran and Hill (2019), the results revealed that the levels of perfectionism have linearly increased, with recent generations perceiving that cultural changes for competitive individualism are more demanding to be perfect.

In educational settings, perfectionism represents an important issue because it has strong relations with achievements (Osenk et al., 2020). More than that, educational context sustains perfectionistic behaviors with both positive outcomes, when we refer to positive perfectionism, such as endorsement of mastery goals for teaching, high level of job satisfaction, flow experience during teaching, and proactive coping (Shim et al., 2020; Samfira and Paloş, 2021) but also negative ones, when we refer to negative or neurotic perfectionism, such as burnout, anxiety, academic procrastination, exhaustion, depressed mood, performance, and work-avoidance goal orientation (Cupido, 2018; Shirazizadeh and Karimpour, 2019; Horan et al., 2021; Serdar et al., 2021; Kilmen, 2022).

Ellis (2019a) mentioned that acceptance occurs when individuals think about themselves and others as imperfect, fallible individuals, who make mistakes. Continuing in the direction of acceptance, Ellis (2019a) sustains that unconditional self-acceptance allows individuals to seek excellence or others’ approval not because of over-generalized needs, but to gratify personal desires. The strong relationship between perfectionism and acceptance is highlighted by Jibeen (2017) who claims that the distinction between conditional and unconditional self-acceptance reflects the distinction sustained by Hamachek (1978) between normal perfectionism, who strive for excellence without any negative effects and neurotic perfectionism, who need acceptance from others.

Translated the relationship between self-acceptance and perfectionism into the educational context, teachers’ self-acceptance implies not only their acceptance as fallible or imperfect persons but also the acceptance of their students, as they also have the right to make mistakes. The acceptance of their negative/imperfect school experiences, according to the principles proposed by Ellis (2019a) supports a strong connection between unconditional self-acceptance, unconditional other-acceptance, and unconditional life-acceptance. The association between teachers’ unconditional self-acceptance and perfectionism was addressed in previous research (Bingöl and Batik, 2019; Samfira and Sava, 2021), showing a negative correlation between unconditional self-acceptance and perfectionism. The more teachers accept themselves as they are, the less perfectionistic behaviors they will manifest. According to the model developed by Ryff and Keyes (1995), this approach has a positive contribution to teachers’ well-being.

### 1.4 Teachers’ pupil control ideology

Educational research has presented that teacher-student relationships are strongly connected with students’ academic achievements, school engagement, prosocial behaviors, and attitudes toward school (Lippard et al., 2018; Longobardi et al., 2021). Students who have positive experiences with their teachers are able to trust and cooperate with them, can be engaged and persistent in challenging tasks, and feel safe in educational activities (Wanders et al., 2020). Perceiving their teachers as warm and caring people facilitates students’ well-being and appreciation of their teachers (Wentzel, 2022). Although representing a classic concept, pupil control ideology is still valid in education (Conriquez, 2020; Samfira and Sava, 2021) and arouses interest.

Pupil control ideology strongly influences teacher-student relationships (Conriquez, 2020), so teachers will manage their classroom order and students’ behavior according to their control ideologies (Ding and Wang, 2018). Pupil control ideology was defined by Willower et al. (1967), who developed the concept as being the teachers’ beliefs about students’ control in classrooms and schools. This ideology ranges along a continuum, from a humanistic orientation (low scores) to a custodial one (high scores). Teachers with a custodial view consider pupils’ misbehavior disrespectful, and therefore, their relationships with students must be autocratic and hierarchical, strict discipline is considered a key to success. Their status is used as a tool to control and manage the classroom (Rideout and Morton, 2010). Custodial teachers show less democratic attitudes and behaviors in the classroom (Demir and Pismek, 2018). Certain researchers (Ding and Wang, 2018) consider custodial pupil control very harmful to children. On the other hand, teachers with a humanistic view are more inclined to consider that pupils have different needs and their relationship with students is warm with open communication. Humanistic teachers develop a democratic environment in the classroom, where students are asked to take an active role in the learning process and to take responsibility for their decisions (Rideout and Morton, 2010). Also, teachers prone to humanistic ideology are more focused on developing soft skills and those competencies considered to be necessary to succeed in their personal and professional life (Ding and Wang, 2018). But this humanistic ideology could lead, in different schools, to their marginalization by the other teachers with a more custodial vision (Giannakaki and Batziakas, 2016). Even though it is a relatively old concept, we find current recommendations for organizing training programs in schools for teachers towards a more humanistic view (Gnanarajan et al., 2020).

To our knowledge, no research has focused on the relationship between the teacher’s pupil control ideology and unconditional self-acceptance, except the study conducted by Samfira and Sava (2021), who showed a negative correlation between these two concepts. According to their results, accepting themselves and their pupils as they are, teachers accept that the world is a complex one and some things or events are sometimes out of their control, especially in the classroom.

To our knowledge, the present study represents the first research on the effectiveness of an REBT intervention in increasing unconditional self-acceptance for in-service teachers. Instead, we identify other interventions (e.g., acceptance and commitment therapy) to increase educators’ self-acceptance (Barida and Widyastuti, 2019).

## 2 The present study

The present research aimed to examine the effectiveness of a cognitive-behavioral intervention with specific REBT techniques on the level of unconditional self-acceptance of primary, secondary, and high school teachers, as well as the robustness of these achievements during follow-up. We assume that *applying specific REBT techniques will significantly improve the teachers’ unconditional self-acceptance scores (USAQ score) in the experimental group compared to the control group, which receives no intervention* (Hypothesis 1a). Also, we assume that *the improvements gained through intervention are maintained over time, six months follow-up* (Hypothesis 1b).

Second, due to the close relationships between unconditional self-acceptance, perfectionism, and pupil control ideology sustained in the previous study conducted by Samfira and Sava (2021), we aim to investigate if teachers’ unconditional self-acceptance will influence the teachers’ level of pupil control ideology and perfectionism. Increasing the level of unconditional self-acceptance will most likely impact the perfectionism level, as people who develop higher levels of unconditional self-acceptance tend to be more tolerant towards mistakes, therefore decrease their level of perfectionism. Likewise, people who decrease their levels of perfectionism, including decreasing the high expectations they put on others (pupils) are more likely to accept or tolerate pupils misbehaviors, and therefore, less likely to adopt a custodial view when interacting with pupils.

Therefore, we hypothesized that *increasing the teachers’ level of unconditional self-acceptance through REBT intervention will lead to lower levels of perfectionism and a less authoritarian ideology* (Hypothesis 2).

**Figure 1:**
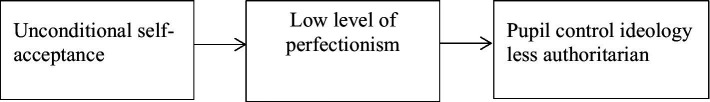
A conceptual and operational analysis of the relationship between unconditional self-acceptance, perfectionism, and pupil control ideology.

Addressing these hypotheses is relevant for both theoretical and practical reasons. Increasing teachers’ level of self-acceptance by applying a cognitive-behavioral intervention will directly contribute to the existing literature in this important domain and indirectly to a more positive learning environment in the classroom and teachers’ well-being. The logic of obtaining this indirect effect is represented conceptually in Figure 1.

The practical reasons are to present an applicable model to help teachers understand these principles of unconditional self-acceptance and apply them to their well-being. As a possible consequence of this new, more rational view, teachers could develop the unconditional acceptance of others (e.g., their students, parents, colleagues) due to its positive outcomes, especially for their students, who need an unconditional teacher to develop and maintain a flourishing relationship (Venet, 2019). In his research, Bernard M (2020) sustains the need to teach and promote self-acceptance, and we consider that unconditional self-acceptance teachers are the most indicated adults to do this because, as Sezer et al. (2020, p. 176) sustain, teachers are seen as “social engineers who laid the foundations of society.”

Another practical aspect is group intervention, which offers more opportunities for teachers to interact with colleagues and share their common experiences than individual intervention (Chenoweth et al., 2016). The intervention could be integrated into the Personal and Professional courses to help in-service teachers to understand and increase their unconditional self-acceptance and to decrease their perfectionistic beliefs and authoritarian ideology. These new gains will help them to have a positive mental state (well-being) and a better relationship with their students, colleagues, and principals.

## 3 Materials and methods

We followed TREND reporting guidelines, the standard for nonrandomized/quasi-experimental study designs (Haynes et al., 2021).

### 3.1 Participants

The participants of the study were 100 in-service teachers, with 50 teachers in the experimental group and 50 teachers in the control group. Participants were primary, secondary, and high-school teachers. First, the researchers determined the adequate sample size, for an effect size of 0.80 and a statistical power of 0.80, and the results claimed that 51 participants were needed for every experimental condition. A comparison of the experimental and control group variables revealed no significant differences for some variables such as the number of participants (experimental: 50, control: 50), gender (experimental: 82% females, control: 84% females), and age (experimental: M = 40.10; SD = 7.58, control: M = 44.40; SD = 8.37). Significant differences were observed for variables such as school level (experimental: 14% primary school teachers, control: 42% primary school teachers), teaching experience (experimental: M = 15.36; SD = 8.95, control: M = 21.67; SD = 9.16), and environment (experimental: 30% urban, control: 54% urban).

### 3.2 Procedure

The eligibility criteria for the teachers’ selection were the school level - secondary school teachers – regardless of their teaching experience and the school location - from Timisoara city (Romania) or peri-urban - for a better adaptation to the teachers’ schedule. Finally, there were accepted even primary school teachers, due to the interest from their part and the principals. As a method of recruitment, we opted for self-selection. In the first stage there was a discussion with the school principal about the main scope of the intervention program and to ask permission. In the second stage, the principal transmitted the information about the intervention/research to all teachers for self-selection. After selecting the teachers who agreed to be part of the experimental group, we proceeded to recruit the teachers for the control group who were selected from different schools than the experimental group, to avoid group contamination. All teachers, from the experimental and control group participated in the study voluntarily, without any financial remuneration.

Concerning ethical considerations, the research was approved by Research and Ethics Committee from West University from Timisoara, being under the 1964 Helsinki Declaration and its later amendments or comparable ethical standards. All the teachers who accepted to participate in this research signed at the recruitment stage of the research, an Informed Consent Form, according to the Ethical standards in research with human subjects. Also, they were assured that could give up their study whenever they want, without any negative consequences. The three questionnaires were administered individually, in a paper-and-pencil format.

### 3.3 Measures

#### 3.3.1 Unconditional self-acceptance questionnaire

We measured teachers’ unconditional self-acceptance with *The Unconditional Self-Acceptance Questionnaire (USAQ)*, a 20-item measure, using a seven-point Likert scale, from almost always false to almost always true. The scale was developed by Chamberlain and Haaga (2001) in line with the REBT perspective. A high score reflects a high level of unconditional self-acceptance. Sample item includes *“I believe that I am worthwhile simply because I am a human being” and “My sense of self-worth depends a lot on how I compare with other people.”* The higher score indicates a higher level of unconditional self-acceptance. The USAQ scale reliability is 0.72 (Chamberlain and Haaga, 2001). The scale was translated from English into Romanian by the authors and then independently back-translated by a third translator. The scale was previously used to assess Romanian teachers’ unconditional self-acceptance by Samfira and Sava (2021).

#### 3.3.2 Pupil control ideology scale

We measured teachers’ pupil control ideology with the *Pupil Control Ideology Scale (PCI)*, a 20-item measure on a five-point Likert scale, from strongly disagree to strongly agree. The scale was developed by Willower et al. (1967). A high score reflects a more custodial orientation and a low one reflects a more humanistic orientation. Sample items include *“It is more important for pupils to learn to obey rules than that they make their own decisions”* and *“Teachers should consider revision of their teaching methods if these are criticized by their pupil”* (reversed). The PCI scale reliability is 0.91 (Willower et al., 1967). The scale was translated from English into Romanian by the authors and then independently back-translated by a third translator.

#### 3.3.3 Perfectionism inventory scale

We measured teachers’ perfectionism with *Perfectionism Inventory Scale (PI),* a 59-item measure, using a five-point Likert scale, from totally disagree to totally agree. The scale was developed by Hill et al. (2004). The sub-scales of the PI are: Concern over Mistakes, High Standards for Others, Need for Approval, Organization, Planfulness, Perceived Parental Pressure, Rumination, and Striving for Excellence. Sample item includes *“To me, a mistake equals failure”* (Concern over Mistakes) and *“I have little tolerance for other people’s careless mistakes”* (high standards for others). The reliability coefficient for the overall level of perfectionism was 0.91 (Hill et al., 2004). The scale was adapted for Romanian teachers by Samfira and Maricuţoiu (2021).

### 3.4 Intervention

The aim of the intervention was to teach the participants of the experimental group the REBT principles of self-acceptance. The objectives were (1) to increase the teachers’ level of unconditional self-acceptance and (2) by accepting oneself, teachers will more easily accept others (pupils). Theoretical perspectives underlying the intervention program were based on two reference books: Ellis and Dryden (1997) - the chapter “Teaching the Principles of Unconditional Self-Acceptance in a Structured Group Settings” and Ellis (1998) - the chapters “Using Unconditional Self-Acceptance” and “Using Unconditional Acceptance of Others to Control Your Anxiety.” The intervention focused on teaching the following topics: the principles of unconditional self-acceptance, self-esteem, the ABC framework of REBT, irrational ego beliefs in the educational context, the zig-zag technique, tape-recorded disputing, the rational-emotive imagery, and the shame-attacking exercises. The researchers organized the exercises related to these topics, as well as the homework following the specifics of the participants’ profession (for educational context). The organization of the program and the content of the homework for each session were supervised by an expert in REBT techniques, from the West University of Timisoara.

In experimental groups, the intervention was delivered face-to-face. The participants in the experimental groups were organized according to their schedules. This aspect has influenced the group size (16–18 teachers). The intervention was delivered in all groups by the first author with Ph.D. in psychology (educational psychologist), with experience in career counseling. Before de intervention begins, the researcher had six meetings with the REBT expert, to discuss the main difficulties in applying REBT technics in the educational area. Also, the same REBT expert mentored the researcher during the period of interventions, to help her in managing challenging situations.

The researcher delivered the intervention in the schools, in the classrooms, to be as easy as possible for the teachers, who ended their classes or were going to have classes. For each group, the established schedule was maintained (day and hour) and the same classroom was used for all meetings. The researcher arrived at the school 30 min before every meeting, to have enough time to prepare the presentation and the incentives (sweets, juices, and mineral water). The punctuality was respected by every participant. The intervention to increase unconditional self-acceptance was administered for 6 weeks. Every session was held weekly and lasted between 90 min, in groups with 5 teachers and 120 min in groups with 12 and 15 teachers. The time was adapted to the group size, to have enough time to help every teacher.

The subjects from the control groups were not subjected to any kind of intervention program. The teachers filled in the same set of questionnaires in the same period and in the classroom, as the teachers in the experimental group, to respect the same conditions as the experimental group. Out of the total of 52 teachers who accepted to participate in the intervention to increase the level of unconditional self-acceptance, 2 of them did not attend at least four sessions and were excluded. The total number of the experimental group remains at 50. To increase compliance and adherence of the group, teachers who attended the minimum number of sessions (4/6) received a Certificate of attendance, for their annual self-assessment. The deliverer has mentioned this aspect from the first meeting, for increasing their motivation to participate. Also, the teachers were offered in each module, sweets, juices, and mineral water.

## 4 Results

The research used a mixed model ANOVA 2 (Group: intervention vs. control) × 3 (Time: baseline vs. post-test vs. 6-month follow-up), with Group as a between-subjects factor and Time as a within-subjects factor. The main outcome is unconditional self-acceptance (the USAQ score). The interaction effect indicated mixed findings. There was a significant interaction between the group and time for the multivariate test, Wilks Lambda = 0.93, *F* (2, 94) = 3.60, *p* = 0.03, partial *η*^2^ = 0.07, and a marginally non-significant interaction effect for the univariate test - *F* (2, 190) = 2.83, *p* = 0.06, partial *η*^2^ = 0.03. These inconsistent findings are most likely due to low statistical power in testing the interaction effect, as *post-hoc* pairwise comparisons using the Bonferroni correction support the interaction effect. In the intervention group, there is a statistically significant increase in the USAQ score from the pre-test to the post-test (*p* < 0.001), which remains significant at the 6-month follow-up (*p* < 0.01). Meanwhile, there were no statistically significant differences in the USAQ scores in the control group, either from pre-test to post-test (*p* = 1.00) or from pre-test to follow-up (*p* = 0.36). Likewise, there were no statistically significant differences in the USAQ scores between post-test and follow-up, neither in the intervention group (*p* = 1.00) nor in the control group (*p* = 0.83; Table 1).

**Table 1:** Mean scores and standard deviations of outcome measures at pre-test, post-intervention, and 6-month follow-up assessment for USAQ (primary outcome) as well as for PCI and PI (secondary outcomes).

Measures/Time	Experimental group			Control group		
	N	Mean	SD	N	Mean	SD
*PCI*						
Pre-test (T1)	50	62.74	8.72	50	62.66	8.59
Post-test (T2)	50	60.18	9.70	50	62.24	9.15
Follow-up (T3)	50	61.22	9.23	47	61.77	9.41
*PI*						
Pre-test (T1)	50	183.30	32.46	50	168.80	28.81
Post-test (T2)	50	178.48	30.78	50	168.82	29.43
Follow-up (T3)	50	176.82	34.64	47	169.77	24.80
*USAQ*						
Pre-test (T1)	50	84.18	10.53	50	92.47	9.13
Post-test (T2)	50	90.16	11.19	50	93.51	9.20
Follow-up (T3)	50	89.32	11.99	47	95.09	14.15

In the case of secondary outcomes, there was no statistically significant interaction effect either for the perfectionism level – *F* (2, 190) = 1.21, *p* = 0.30, partial *η*^2^ = 0.01, or for the level of pupil control ideology – *F* (2, 190) = 1.12, *p* = 0.33, partial *η*^2^ = 0.01. For investigating the association between unconditional self-acceptance, perfectionism, and pupil control ideology, we computed change scores (i.e., from pre-test to post-test). We found a statistically significant negative association between the magnitude of change in the USAQ score and the magnitude of change in the overall perfectionism – *r* (98) = −0.33, *p* < 0.001, two-tailed test. Participants who learned to a higher extent how to accept themselves unconditionally decreased to a higher degree their perfectionism level. Likewise, there was a statistically significant positive association between the magnitude of change in the perfectionism level and the degree of change in the pupil control ideology – *r* (98) = 0.27, *p* < 0.01, two-tailed test. Participants who reduced their perfectionism level to a higher extent were also less willing to endorse custodial beliefs and more willing to endorse humanistic beliefs on pupil control. Similar results were obtained when conducting the analyses on change scores from pre-test to 6-month follow-up measurements instead of pre-test to post-test change scores, which highlights the findings’ robustness. To test whether the magnitude change in the USAQ scores indirectly affected pupil control ideology via the perfectionism level, we used Process Analysis v. 4.1 (Hayes, 2022). The indirect effect of change in USAQ scores on change in PCI scores was marginally not statistically significant both when looking at change scores from baseline to post-intervention – standardized indirect effect of −0.10 [−0.23 to 0.00] and when looking at change scores from baseline to follow-up – standardized indirect effect of −0.10 [−0.22 to 0.00].

These results demonstrate that the effectiveness of the intervention is successful in increasing the level of unconditional self-acceptance, our primary dependent variable, and the result is stable across the 6-month follow-up.

## 5 Discussion

The purpose of the present research was first to examine the effectiveness of a cognitive-behavioral intervention on the level of unconditional self-acceptance for school teachers, as well as the robustness of these achievements during follow-up (6 months later). Based on the research results, we concluded that the intervention designed to support and increase the teachers’ level of unconditional self-acceptance was efficient in helping teachers to internalize the philosophy of self-acceptance, accepting themselves as fallible and imperfect human beings, through a process of understanding how their beliefs and thoughts process influences their emotional and behavioral well-being. These findings are in line with other studies, which have shown that REBT interventions are efficient in increasing the level of unconditional self-acceptance in different contexts: educational (Pasaribu and Zarfiel, 2019), sport (Knapp et al., 2023), clinical (Artiran and DiGiuseppe, 2022), and the general population (Crișan et al., 2022). There are no studies analyzing the effectiveness of interventions to increase teachers’ unconditional self-acceptance.

Relating to the follow-up, the results showed that there were no significant changes from post-test to follow-up. This outcome highlights that the teachers’ unconditional self-acceptance achieved at the post-test remained stable over time, in this case, 6 months. This result is a hopeful one because demonstrates that teachers could maintain in time their rational beliefs about themselves, their children, and the teaching profession.

For the control group, the results showed that there were no significant changes from pre-test to post-test. This result reflects what DiGiuseppe et al. (2002) sustain that “achievement of unconditional self-acceptance (USA) is a difficult, though possible task, that requires time and commitment” (p. 229). Also, Bernard M (2020) recommended, that teachers and principals must be trained on how to improve their self-acceptance and combat their self-depreciation. The same thing could be said about the absence of any statistically significant differences in the USAQ scores between post-test and follow-up, highlighting the idea that unconditional self-acceptance is developed only through coaching (Palmer and Williams, 2021). Even for the individuals who attend intervention programs to change their irrational beliefs, it is quite difficult, due to the “biological tendency of humans to behave irrationally” (Ellis, 1976, p. 5). These findings support the first hypothesis (1a and 1b) of the present research, about existing a significant improvement in teachers’ unconditional self-acceptance scores (USAQ score) from the experimental group compared to the control group, which did not receive any intervention and about maintaining these changes over time (6 months later in this case).

In the case of secondary outcomes, the results showed that applying an REBT intervention to increase teachers’ unconditional self-acceptance does not have statistically significant consequences in decreasing teachers’ perfectionism and teachers’ custodial/authoritarian ideology. Even though many studies recommended REBT techniques to reduce perfectionistic beliefs and the theory sustained that by adopting an unconditional self-acceptance philosophy the individuals will accept themselves as fallible and imperfect persons (Cohen, 2019), an REBT intervention with specific tasks for increasing unconditional self-acceptance has not enough impact for teachers to reduce their level of perfectionism. The main reason could be related to the specific of the teaching profession which, due to the high standards promoted, encourages perfectionistic tendencies in teachers (Cupido, 2018; Shim et al., 2020). Student grades rank schools and for this reason, many principals put great pressure on the teachers to get very good results from their students. In this stressful context, teachers are somewhat forced to become perfectionists. But, at the same time, recent studies (Hill, 2022) sustain that school has also the role to help students to cope with high expectations, pressure, and unhealthy perfectionistic standards.

Analyzing the result, which sustains that there is no statistically significant effect of an REBT intervention to increase teachers’ unconditional self-acceptance on decreasing teachers’ pupil control ideology (the custodial/authoritarian view), we could sustain what other researchers concluded after applying a specific intervention to decrease teachers’ pupil control ideology – that changes in the direction of reduction the authoritarian view is easier to describe than to do, and are often unsuccessful (Hoy and Miskel, 1978). Even though the statement is very old, it remains current because no recent studies have been identified in the direction of pupil control ideology reduction. Similar to the previous outcomes (Samfira and Sava, 2021), admitting that there is a negative correlation between teachers’ unconditional self-acceptance and pupil control ideology (the custodial/authoritarian view), the intention to increase the level of unconditional self-acceptance does not imply automatically significant changes in teachers’ pupil control ideology (decreasing the authoritarian view). Both results align with other papers that applied REBT intervention to increase unconditional self-acceptance but without significantly affecting all variables included in their research (Crișan et al., 2022).

Accepting that some results were not statistically significant, as in the case of perfectionism and control ideology, we could not omit the close relationship that exists between the unconditional self-acceptance, perfectionism, and pupil control ideology sustained by previous studies (Samfira and Sava, 2021). Consequently, we explored the association between unconditional self-acceptance, perfectionism, and pupil control ideology and computed change scores (i.e., from pre-test to post-test). We opted for change scores because being quasi-experimental research, the two groups were not randomized and they were not equivalent in their mean scores in the pre-test (T1) and the mean scores for USAQ were higher in the control group than in the experimental group. Analyzing the data, we identified a statistically significant negative association between the magnitude of change in the USAQ score and the magnitude of change in the overall perfectionism scores. These results highlight what Cohen (2019) claims, that being an unconditional self-accepting person means, among others, “being comfortable with your imperfections, letting go your demand for perfection, understanding that making mistakes … does not diminish your value as a person” (p. 31). A significant result was also found for pre-test to 6-month follow-up measurements, the result highlighting the robustness of our findings.

Consequently, we found a statistically significant negative association between the change in the perfectionism score and the change in the pupil control ideology from the pre-test to the post-test. These results could be interpreted that teachers who decrease their perfectionistic level, attending the intervention with REBT techniques, are more willing to approve a humanistic belief in pupil control. This result is in line with other studies (Samfira and Sava, 2021), which found that teachers who adopt a more humanistic view of pupil control ideology, have lower levels of perfectionism. A similar statistically significant result was found for pre-test to 6-month follow-up measurements, sustaining our findings’ robustness. Maybe teachers have had positive experiences regarding unconditional self-acceptance, which helped them to accept mistakes and imperfections in their lives and also in their students’ behaviors (a lower level of perfectionism), which consequently lead to a more humanistic ideology in relationships with their students. Adopting this humanistic ideology, more flexible, more open, and more closed to the children, teachers could help themselves to cooperate with other teachers, to meet the different needs of their students, become the educators they wish to be, and also could help the students through developing a positive teacher-student relationship (Allender and Sclarow-Allender, 2015).

To sum up the discussion of results, this is the first research that applies an REBT intervention to increase in-service teachers’ unconditional self-acceptance. There are few studies on children (Bernard et al., 2013) and university students (Pasaribu and Zarfiel, 2019), but none on teachers. Another strength point is related to the success of the intervention. The intervention has received accreditation from the Ministry of Education to be part of the professional development programs for teaching staff. It could be used as a prevention program as teachers often are confronted with environments where they could experience high levels of anxiety (Liu et al., 2022). The third strength point is represented by the length of the intervention (6 weeks) compared with other interventions on unconditional self-acceptance, which took place for only 7 days (Crișan et al., 2022). As DiGiuseppe et al. (2007) sustain, achievement of unconditional self-acceptance requires time. The fourth strength point is represented by the participants, who were in-service teachers, with experience between 1 and 40 years in dealing with students, not pre-service teachers, who are supposed to be more idealistic in their beliefs.

### 5.1 Limitations and future directions

Similar to any other research, this paper has limitations. The major limitation of the present research was the non-random allocation of the teachers into the experimental and control groups. Due to their busy schedules, teaching in two or three schools, the randomization of the teachers was impossible. Because of this there were differences in the composition of the two groups. In the experimental group, there were mostly high school and middle school teachers than in the control group, the latter including more primary school teachers than the experimental group. This could be the main reason for the differences in unconditional self-acceptance pre-test scores, the unconditional self-acceptance baseline values being higher in the control group than in the experimental group.

Future studies with a more robust design, such as randomizing teachers into experimental and control conditions, are welcomed to replicate the current results obtained from a quasi-experimental approach. This will lead to a more balanced distribution of primary, secondary, and high school teachers in the experimental and control groups to have an overview of their beliefs about teacher-student relationships.

Another recommendation is to introduce REBT principles in teacher education programs, for personal and professional development, to help future teachers to become more rational teachers and to be able to apply these principles in school settings or to prevent work-life conflict (Ogakwu et al., 2022, 2023).

## 6 Conclusion

The findings of this research indicated that an REBT intervention designed to increase the teachers’ level of unconditional self-acceptance had success, with teachers’ mean scores significantly increasing from pre-test to post-test, after 6 weeks. The increased scores remained stable across the 6-month follow-up, these findings showing that teachers internalized the unconditional self-acceptance philosophy and applied it. In the control group, there were no statistically significant differences in the USAQ scores, neither from pre-test to post-test nor from pre-test to follow-up. These results show us that a specific intervention is necessary when there is an intention to increase an individual’s unconditional self-acceptance.

Another important result is the association between changes in the scores for unconditional self-acceptance, perfectionism, and pupil control ideology in the expected directions. As the level of unconditional self-acceptance increases, there is a reduction in the perfectionism level. Likewise, as perfectionism decreases among teachers, they are less prone to endorse custodial views on their students. However, the link between these constructs is relatively weak despite these correlational findings. Therefore, increasing the levels of unconditional self-acceptance through an REBT intervention will not automatically decrease their levels of perfectionism and custodial/authoritarian ideology. These findings could be interpreted that it is necessary either to improve the intervention with specific techniques for perfectionism and pupil control ideology to decrease their perfectionistic beliefs and custodial ideology.

Overall, the 6-week REBT face to face group intervention provides an efficient way in which people could increase their level of unconditional self-acceptance, a gain of great value for their ability to cope with stressful events in educational settings. Despite some evidence for additional indirect positive outcomes, such as a lower level of perfectionism, and a friendlier interaction with students, additional 1 or to 2 sessions should be introduced in a future version of the program to confront more directly the perfectionism of teacher that often accompanies low levels of unconditional self-acceptance.

## Data availability statement

The raw data supporting the conclusions of this article will be made available by the authors, without undue reservation.

## Ethics statement

The studies involving humans were approved by West University of Timisoara Institutional Review Board (Research Ethics Committee). The studies were conducted in accordance with the local legislation and institutional requirements. The participants provided their written informed consent to participate in this study.

## Author contributions

ES has contributed to the conceptualization, coordinated the collection of the data, applied the intervention, wrote the original draft, and edited the final manuscript. FS has contributed to the conceptualization, design, methodology, writing, and reviewing of the final manuscript. All authors contributed to the article and approved the submitted version.

## References

[ref1] Aftab M. Khatoon T. (2013). Influence of gender, types of school and occupational stress on pupil control ideology of secondary school teachers in India. J. Educ. Pract. 4, 64–72.

[ref2] Akaki M. Kobayashi N. Shirasaka S. Ioki M. (2020). The effect of a method to enhance self-acceptance and acceptance of others through collaborative Team’s role recognition. Int. J. Ser. Knowl. Manage. 4, 76–95. doi: 10.52731/ijskm.v4.i1.511

[ref3] Allender J. S. Sclarow-Allender D. (2015). Humanistic Teacher: First the Child, then Curriculum. New York, NY: Routledge.

[ref4] Andronikos P. N. (2021). The Contribution of Self-Esteem, Self-Compassion, and Self-Acceptance/Self-Condemnation in Predicting Psychopathology and Well-being. [Doctoral Thesis]. New York (NY): St. John's University.

[ref5] Artiran M. (2019). A Cross-Cultural Redefinition of Rational Emotive and Cognitive Behavior Therapy: From the West to the Middle East. New York, NY: Routledge.

[ref6] Artiran M. DiGiuseppe R. (2022). Rational emotive behavior therapy compared to client-centered therapy for outpatients: a randomized clinical trial with a three months follow up. J. Ration. Emot. Cogn. Behav. Ther. 40, 206–233. doi: 10.1007/s10942-021-00408-0

[ref7] Asaloei S. I. Wolomasi A. K. Werang B. R. (2020). Work-related stress and performance among primary school teachers. Int. J. Eval. Res. Educ. 9, 352–358. doi: 10.11591/ijere.v9i2.20335

[ref8] Barida M. Widyastuti D. A. (2019). Acceptance and commitment therapy (ACT) to improve educators self-acceptance of children with special needs. KONSELI: Jurnal Bimbingan dan Konseling. 6, 117–124. doi: 10.24042/kons.v6i2.4701

[ref9] Beeghly E. (2021). What's wrong with stereotypes? The falsity hypothesis. Soc. Theory Pract. 47, 33–61. doi: 10.5840/soctheorpract2021112111

[ref11] Bernard M. E. (2016). Teacher beliefs and stress. J. Ration. Emot. Cogn. Behav. Ther. 34, 209–224. doi: 10.1007/s10942-016-0238-y

[ref12] Bernard M. (2020). “Self-acceptance: REBT as the psychological armor that protects children and adolescents” in Rational-Emotive and Cognitive-Behavioral Approaches to Child and Adolescent Mental Health: Theory, Practice, Research, Applications. eds. Bernard M. Terjesen M. D. (Berlin: Springer, Cham), 223–240.

[ref13] Bernard M. E. (2020). Self-Acceptance: The Foundation of Mental Health and Wellbeing. Available at: https://www.youcandoiteducation.com.au/wp-content/uploads/2019/11/Self-Acceptance-TheFoundation-Mental-Health-Wellbeing.pdf (Accessed January 24, 2023).

[ref14] Bernard M. E. Vernon A. Terjesen M. Kurasaki R. (2013). “Self-acceptance in the education and counseling of young people” in The Strength of Self-Acceptance: Theory, Research, and Practice. ed. Bernard M. E. (New York: Springer), 155–192.

[ref15] Bingöl T. Y. Batik M. V. (2019). Unconditional self-acceptance and perfectionistic cognitions as predictors of psychological well-being. J. Educ. Train. Stud. 7, 67–75. doi: 10.11114/jets.v7i1.3712

[ref16] Bonde E. H. Fjorback L. O. Frydenberg M. Juul L. (2022). The effectiveness of mindfulness-based stress reduction for school teachers: a cluster-randomized controlled trial. Eur. J. Pub. Health 32, 246–253. doi: 10.1093/eurpub/ckab223, PMID: 35142355 PMC8975540

[ref17] Brady J. Wilson E. (2022). Comparing sources of stress for state and private school teachers in England. Improv. Sch. 25, 205–220. doi: 10.1177/13654802211024758

[ref18] Candeias A. Galindo E. Calisto I. Borralho L. Reschke K. (2021). Stress and burnout in teaching. Study in an inclusive school workplace. Health Psychol. Rep. 9, 63–75. doi: 10.5114/hpr.2020.100786 38084117 PMC10694697

[ref19] Chadha N. J. Turner M. J. Slater M. J. (2019). Investigating irrational beliefs, cognitive appraisals, challenge and threat, and affective states in golfers approaching competitive situations. Front. Psychol. 10:2295. doi: 10.3389/fpsyg.2019.02295, PMID: 31649600 PMC6795749

[ref20] Chamberlain J. M. Haaga D. A. (2001). Unconditional self-acceptance and psychological health. J. Ration. Emot. Cogn. Behav. Ther. 19, 163–176. doi: 10.1023/A:1011141500670

[ref21] Chenoweth L. Stein-Parbury J. White D. McNeill G. Jeon Y. H. Zaratan B. (2016). Coaching in self-efficacy improves care responses, health, and well-being in dementia carers: a pre/post-test/follow-up study. BMC Health Serv. Res. 16, 1–16. doi: 10.1186/s12913-016-1410-x, PMID: 27146060 PMC4855813

[ref22] Cohen E. D. (2019). Making Peace with Imperfection: Discover Your Perfectionism Type, End the Cycle of Criticism, and Embrace Self-Acceptance. Oakland, CA: New Harbinger Publications.

[ref23] Conriquez J. (2020). The Relationship Between Teacher Beliefs, Classroom Management, and Teacher-Student Relationships. [Dissertation Thesis]. [California (CA)]: California State University, San Bernardino.

[ref24] Corcoran R. P. O'Flaherty J. (2022). Social and emotional learning in teacher preparation: pre-service teacher well-being. Teach. Teach. Educ. 110:103563. doi: 10.1016/j.tate.2021.103563

[ref25] Crișan S. Canache M. Buksa D. Nechita D. (2022). A comparison between self-compassion and unconditional self-acceptance: interventions on self-blame, empathy, shame-, guilt-proneness, and performance. J. Ration. Emot. Cogn. Behav. Ther. 41, 64–80. doi: 10.1007/s10942-022-00451-5

[ref26] Cupido C. (2018). Music performance anxiety, perfectionism and its manifestation in the lived experiences of singer-teachers. Muziki. 15, 14–36. doi: 10.1080/18125980.2018.1467367

[ref27] Curran T. Hill A. P. (2019). Perfectionism is increasing over time: a meta-analysis of birth cohort differences from 1989 to 2016. Psychol. Bull. 145, 410–429. doi: 10.1037/bul0000138 29283599

[ref28] Demir S. B. Pismek N. (2018). A convergent parallel mixed-methods study of controversial issues in social studies classes: a clash of ideologies. Educ. Sci. Theor. Pract. 18, 119–149. doi: 10.12738/estp.2018.1.0298

[ref29] DiGiuseppe R. Doyle K. A. Rose R. D. (2002). “Rational-emotive behavior for depression: achieving unconditional self-acceptance” in Comparative Treatments of Depression. eds. Reinecke M. A. Davison M. R. (New York, NY: Springer), 220–248.

[ref9001] DiGiuseppe R. Doyle K. A. Rose R. D. (2007). Rational-emotive behavior therapy for depression: achieving unconditional self-acceptance. in Depression: A Practitioner’s Guide to Comparative Treatments. eds. Reinecke M. A. Davison M. R. (New York, NY: Springer), 220–248.

[ref30] Dike I. C. Onyishi C. N. Adimora D. E. Ugodulunwa C. A. Adama G. N. Ugwu G. C.. (2021). Yoga complemented cognitive behavioral therapy on job burnout among teachers of children with autism spectrum disorders. Medicine 100:e25801. doi: 10.1097/MD.0000000000025801, PMID: 34087823 PMC8183729

[ref31] Ding A. C. Wang H. H. (2018). Unpacking teacher candidates’ decision-making and justifications in dilemmatic spaces during the student teaching year. Asia Pac. J. Teach. Educ. 46, 221–238. doi: 10.1080/1359866X.2018.1442916

[ref32] Dryden W. Neenan M. (2020). Rational Emotive Behaviour Therapy: 100 Key Points and Techniques. 3rd Edn. New York, NY: Routledge.

[ref33] Ellis A. (1957). Rational psychotherapy and individual psychology. J. Individ. Psychol. 13, 38–44.

[ref34] Ellis A. (1976). The biological basis of human irrationality. J. Individ. Psychol. 32, 145–168. 993611

[ref35] Ellis A. (1977). “Psychotherapy and the value of a human being” in Handbook of Rational-Emotive Therapy. eds. Ellis A. Grieger R. (New York, NY: Springer), 99–112.

[ref36] Ellis A., (1994). Reason and Emotion in Psychotherapy: A Comprehensive Method of Treating Human Disturbances. New York: Birch Lane Press.

[ref37] Ellis A. (1998). How to Control Your Anxiety Before It Controls You. New York: Citadel Press.

[ref38] Ellis A. (2019a). How to Stubbornly Refuse to Make Yourself Miserable: About Anything-Yes, Anything! London, UK: Little Brown Group.

[ref39] Ellis A. (2019b). “Early theories and practices of rational emotive behavior therapy and how they have been augmented and revised during the last three decades” in Advances in REBT. Theory, Practice, Research, Measurement, Prevention and Promotion. eds. Bernard M. E. Dryden W. (Cam: Springer), 1–21.

[ref40] Ellis A. E. Bernard M. E. (2006). Rational Emotive Approaches to the Problems of Childhood. 2nd Edn. New York, NY: Springer.

[ref41] Ellis A. Dryden W. (1997). The Practice of Rational Emotive Behavior Therapy. 2nd. New York, NY: Springer Publishing Company.

[ref42] Evans A. L. Turner M. J. Pickering R. Powditch R. (2018). The effects of rational and irrational coach team talks on the cognitive appraisal and achievement goal orientation of varsity football athletes. Int. J. Sports Sci. Coach. 13, 431–438. doi: 10.1177/1747954118771183

[ref43] Giannakaki M. S. Batziakas G. (2016). This is a beautiful school. This school is useless!! Explaining disengagement in a Greek vocational school through the examination of teacher ideologies. Res. Postcompulsory Educ. 21, 409–433. doi: 10.1080/13596748.2016.1226585

[ref44] Gkontelos A. Vaiopoulou J. Stamovlasis D. (2021). Teachers’ irrational belief scale: psychometric properties of the Greek version and measurement invariance across genders. Behav. Sci. 11:160. doi: 10.3390/bs11110160, PMID: 34821621 PMC8615129

[ref45] Gnanarajan A. H. Kengatharan N. Velnampy T. (2020). Exploring the prevalence of teachers’ organizational citizenship behaviour and its determinants: evidence from an under-researched cultural milieu. Qual. Res. Educ. 9, 95–123. doi: 10.17583/qre.2020.4531

[ref46] Godin J. (2010). The Effect of the Enneagram on Psychological Well-Being and Unconditional Self-Acceptance of Young Adults. [Dissertation Thesis]. Iowa (IA): Iowa State University.

[ref47] Gran S. (2021). Using NLP (Neuro-Linguistic Programming) Methods in Teaching and Learning: Case Studies on the Potential and Impact of NLP Methods on Learning and Learners. [Doctoral Thesis]. Duisburg, Germany: Universität Duisburg-Essen.

[ref48] Guterman L. (2020). The Progression of Parenting and Childhood Leading to Developing an Understanding of Unconditional Self-Acceptance. [Doctoral Thesis]. Hempstead (NY): Hofstra University.

[ref49] Hamachek D. E. (1978). Psychodynamics of normal and neurotic perfectionism. Psychology 15, 27–33.

[ref50] Hayes A. F. (2022). Introduction to Mediation, Moderation, and Conditional Process Analysis: A Regression-Based Approach. 3rd. New York, NY: Guilford Press.

[ref51] Haynes A. B. Haukoos J. S. Dimick J. B. (2021). TREND reporting guidelines for nonrandomized/quasi-experimental study designs. JAMA Surg. 156, 879–880. doi: 10.1001/jamasurg.2021.0552, PMID: 33825826

[ref52] Hill A. P. (2022). Perfectionism Can Harm Even the Most Talented Student–But Schools Can Make a Difference. The Conversation. Available at: https://ray.yorksj.ac.uk/id/eprint/5833/1/perfectionism-can-harm-even-the-most-talented-student-but-schools-can-make-a-difference-174504 (Accessed 31, March 2023)

[ref53] Hill A. P. Appleton P. R. Mallinson S. H. (2016). Development and initial validation of the performance perfectionism scale for sport (PPS-S). J. Psychoeduc. Assess. 34, 653–669. doi: 10.1177/0734282916651354

[ref54] Hill R. W. Huelsman T. J. Furr R. M. Kibler J. Vicente B. B. Kennedy C. (2004). A new measure of perfectionism: the perfectionism inventory. J. Pers. Assess. 82, 80–91. doi: 10.1207/s15327752jpa8201_13 PMID: 14979837, PMID: 14979837

[ref55] Horan S. Flaxman P. E. Stride C. B. (2021). The perfect recovery? Interactive influence of perfectionism and spillover work tasks on changes in exhaustion and mood around a vacation. J. Occup. Health Psychol. 26, 86–107. doi: 10.1037/ocp0000208, PMID: 32584120

[ref56] Hoy W. K. Miskel C. G. (1978). Educational Administration: Theory, Research and Practice. New York, NY: Random House.

[ref57] Huk O. Terjesen M. D. Cherkasova L. (2019). Predicting teacher burnout as a function of school characteristics and irrational beliefs. Psychol. Sch. 56, 792–808. doi: 10.1002/pits.22233

[ref58] Ifelunni C. O. Ede M. O. Okeke C. I. (2022). Rational emotive intervention for work-family conflict and female primary school teachers’ well-being. Curr. Psychol. 42, 26173–26186. doi: 10.1007/s12144-022-03704-9, PMID: 36124047 PMC9476453

[ref59] Jerrim J. Sims S. Taylor H. Allen R. (2020). How does the mental health and wellbeing of teachers compare to other professions? Evidence from eleven survey datasets. Rev. Educ. 8, 659–689. doi: 10.1002/rev3.3228

[ref60] Jibeen T. (2017). Unconditional self acceptance and self esteem in relation to frustration intolerance beliefs and psychological distress. J. Ration. Emot. Cogn. Behav. Ther. 35, 207–221. doi: 10.1007/s10942-016-0251-1

[ref61] Kidger J. Turner N. Hollingworth W. Evans R. Bell S. Brockman R.. (2021). An intervention to improve teacher well-being support and training to support students in UK high schools (the WISE study): a cluster randomised controlled trial. PLoS Med. 18:e1003847. doi: 10.1371/journal.pmed.1003847, PMID: 34762673 PMC8629387

[ref62] Kilmen S. (2022). Prospective teachers' professional achievement goal orientations, their self-efficacy beliefs, and perfectionism: a mediation analysis. Stud. Educ. Eval. 74:101165. doi: 10.1016/j.stueduc.2022.101165

[ref63] Kim J. Shin Y. Tsukayama E. Park D. (2020). Stress mindset predicts job turnover among preschool teachers. J. Sch. Psychol. 78, 13–22. doi: 10.1016/j.jsp.2019.11.002, PMID: 32178808

[ref64] Knapp S. Miller A. Outar L. Turner M. (2023). Psychological well-being and exercise addiction: the treatment effects of an REBT intervention for females. Psychol. Sport Exerc. 64:102298. doi: 10.1016/j.psychsport.2022.102298, PMID: 37665799

[ref65] Kohn A. (2005). Unconditional teaching. Educ. Leadership 63, 20–24.

[ref66] Lapshin K. (2020). Why Is It Important to Accept Others for They Are. Available at: https://ironyoflife.com/important-to-accept-others-for-who-they-are/ (Accessed 29, 2023)

[ref67] Lippard C. N. La Paro K. M. Rouse H. L. Crosby D. A. (2018). A closer look at teacher–child relationships and classroom emotional context in preschool. Child. Youth. Care. Forum. 47, 1–21. doi: 10.1007/s10566-017-9414-1

[ref68] Liu G. Yan C. Fu J. (2022). Exploring livestream English teaching anxiety in the Chinese context: an ecological perspective. Teach. Teach. Educ. 111:103620. doi: 10.1016/j.tate.2021.103620

[ref69] Longobardi C. Settanni M. Lin S. Fabris M. A. (2021). Student–teacher relationship quality and prosocial behaviour: the mediating role of academic achievement and a positive attitude towards school. Br. J. Educ. Psychol. 91, 547–562. doi: 10.1111/bjep.12378, PMID: 32920835

[ref70] Mitchell R. M. Kensler L. Tschannen-Moran M. (2018). Student trust in teachers and student perceptions of safety: positive predictors of student identification with school. Int. J. Leadersh. Educ. 21, 135–154. doi: 10.1080/13603124.2016.1157211

[ref71] Nwabuko L. O. Eze G. C. Eneh E. C. Okechukwu A. E. Udom I. E. (2020). Effect of rational-emotive adult education intervention on burnout symptoms among primary school teachers in Southeast Nigeria. J. Int. Med. Res. 48:030006051988220. doi: 10.1177/0300060519882204, PMID: 31880181 PMC7607523

[ref72] Obiweluozo P. E. Dike I. C. Ogba F. N. Elom C. O. Orabueze F. O. Okoye-Ugwu S.. (2021). Stress in teachers of children with neuro-developmental disorders: effect of blended rational emotive behavioral therapy. Sci. Prog. 104:00368504211050278. doi: 10.1177/00368504211050, PMID: 34783626 PMC10402289

[ref73] Ogakwu N. V. Ede M. O. Amaeze F. E. Manafa I. Okeke F. C. Omeke F.. (2022). Occupational health intervention for work–life balance and burnout management among teachers in rural communities. J. Community Psychol. 50, 2923–2937. doi: 10.1002/jcop.22806, PMID: 35187678

[ref74] Ogakwu N. V. Ede M. O. Manafa I. F. Okeke C. I. Onah S. O. (2023). Quality of work-life and stress Management in a Rural Sample of primary school teachers: an intervention study. J. Ration. Emot. Cogn. Behav. Ther., 1–27. Advance online publication doi: 10.1007/s10942-022-00494-8

[ref75] Onuigbo L. N. Onyishi C. N. Eseadi C. (2020). Clinical benefits of rational-emotive stress management therapy for job burnout and dysfunctional distress of special education teachers. World J. Clin. Cases 8, 2438–2447. doi: 10.12998/wjcc.v8.i12.2438, PMID: 32607321 PMC7322436

[ref76] Osenk I. Williamson P. Wade T. D. (2020). Does perfectionism or pursuit of excellence contribute to successful learning? A meta-analytic review. Psychol. Assess. 32, 972–983. doi: 10.1037/pas0000942 32718164

[ref77] Palmer S. Williams H. (2021). “Developing self-acceptance through coaching” in Cognitive Behavioural Coaching in Practice. An Evidence Based Approach. eds. Neenan M. Palmer S. (London, UK: Routledge), 99–125.

[ref78] Pasaribu P. E. Zarfiel M. D. (2019). Cognitive behavioral therapy treatment for reducing stress: a case study of self-acceptance in an early adult college student. Adv. Soc. Sci. Educ. Hum. Res. 229, 631–644. doi: 10.2991/iciap-18.2019.54

[ref79] Popov S. (2019). When is unconditional self-acceptance a better predictor of mental health than self-esteem? J. Ration. Emot. Cogn. Behav. Ther 37, 251–261. doi: 10.1007/s10942-018-0310-x

[ref80] Porada K. Sammut S. Milburn M. (2018). Empirical investigation of the relationships between irrationality, self-acceptance, and dispositional forgiveness. J. Ration. Emot. Cogn. Behav. Ther 36, 234–251. doi: 10.1007/s10942-017-0284-0

[ref81] Potter J. H. (2021). Teachers’ Stress, Anxiety, and Depression: What are Special Education Teachers Experiencing? [Doctoral Thesis]. Nacogdoches (TX): Stephen F. Austin State University.

[ref82] Prihadi K. Hui Y. L. Chua M. Chang C. K. (2019). Cyber-victimization and perceived depression: serial mediation of self-esteem and learned-helplessness. Int. J. Eval. Res. Educ. 9, 563–574. doi: 10.11591/ijere.v8i4.20266

[ref83] Rideout G. Morton L. (2010). Pre-service teachers’ beliefs and pupil control ideology: the custodializing practicum. J. Educ. Admin. 48, 64–88. doi: 10.1108/09578231011015421

[ref84] Ryff C. D. Keyes C. L. M. (1995). The structure of psychological well-being revisited. J. Pers. Soc. Psychol. 69, 719–727. doi: 10.1037/0022-3514.69.4.719 7473027

[ref85] Samfira E. M. Maricuţoiu L. P. (2021). Not all perfectionists are as they are assessed: an investigation of the psychometric properties of the perfectionism inventory in the teaching profession. Front. Psychol. 12:624938. doi: 10.3389/fpsyg.2021.624938, PMID: 33643154 PMC7902758

[ref86] Samfira E. M. Paloş R. (2021). Teachers’ personality, perfectionism, and self-efficacy as predictors for coping strategies based on personal resources. Front. Psychol. 12:751930. doi: 10.3389/fpsyg.2021.751930, PMID: 34795619 PMC8593193

[ref87] Samfira E. M. Sava F. A. (2021). Cognitive-behavioral correlates of pupil control ideology. PLoS One 16:e0246787. doi: 10.17605/OSF.IO/A8HGJ, PMID: 33566843 PMC7875416

[ref88] Schellings G. Koopman M. Beijaard D. Mommers J. (2023). Constructing configurations to capture the complexity and uniqueness of beginning teachers’ professional identity. Eur. J. Teach. Educ. 46, 372–396. doi: 10.1080/02619768.2021.1905793

[ref89] Serdar E. Harmandar Demirel D. Demirel M. (2021). The relationship between academic procrastination, academic motivation and perfectionism: a study on teacher candidates. Turk Online J. Educ. Technol. 20, 140–149.

[ref90] Sevim O. Akan D. Yildirim I. (2020). Cognitive constructs of teacher candidates on ideal qualifications of academicians. Int. J. Educ. Lit. Stud. 8, 76–89. doi: 10.7575/aiac.ijels.v.8n.3p.76

[ref91] Sezer S. Karabacak N. Kucuk M. Korkmaz İ. (2020). School administrators’ opinions related to the values that should be gained to classroom teachers through in-service training. Eurasian J. Educ. Res. 20, 1–22. doi: 10.14689/ejer.2020.86.9

[ref92] Shim S. Cho Y. Knapke M. (2020). Perils of perfectionistic concerns among teachers. Psychol. Sch. 57, 1116–1131. doi: 10.1002/pits.22384

[ref93] Shirazizadeh M. Karimpour M. (2019). An investigation of the relationships among EFL teachers’ perfectionism, reflection and burnout. Cogent Educ. 6:1667708. doi: 10.1080/2331186X.2019.1667708

[ref94] Stoeber J. (2017). The Psychology of Perfectionism: Theory, Research, Applications. London, UK: Routledge.

[ref95] Stoeber J. Smith M. M. Saklofske D. H. Sherry S. B. (2021). Perfectionism and interpersonal problems revisited. Pers. Individ. Dif. 169:110106. doi: 10.1016/j.paid.2020.110106

[ref96] Sun J. Wang Y. Wan Q. Huang Z. (2019). Mindfulness and special education teachers' burnout: the serial multiple mediation effects of self-acceptance and perceived stress. Soc. Behav. Personal. 47, 1–8. doi: 10.2224/sbp.8656

[ref97] Toropova A. Myrberg E. Johansson S. (2021). Teacher job satisfaction: the importance of school working conditions and teacher characteristics. Educ. Rev. 73, 71–97. doi: 10.1080/00131911.2019.1705247

[ref98] Trip S. Bora C. H. Roseanu G. McMahon J. (2021). Anger, frustration intolerance, global evaluation of human worth and externalizing behaviors in preadolescence. J. Ration. Emot. Cogn. Behav. Ther. 39, 238–255. doi: 10.1007/s10942-020-00369-w

[ref99] Venet A. S. (2019). Role-clarity and boundaries for trauma-informed teachers. Educ. Considerations. 44, 3–9. doi: 10.4148/0146-9282.2175

[ref100] Vural-Batik M. (2019). The predictive role of homophobia and unconditional self-acceptance on respect of differences in psychological counselor candidates. Int. J. Educ. Methodol. 5, 59–70. doi: 10.12973/ijem.5.1.59

[ref101] Wanders F. H. Dijkstra A. B. Maslowski R. Van der Veen I. (2020). The effect of teacher-student and student-student relationships on the societal involvement of students. Res. Pap. Educ. 35, 266–286. doi: 10.1080/02671522.2019.1568529

[ref102] Wentzel K. R. (2022). Does anybody care? Conceptualization and measurement within the contexts of teacher-student and peer relationships. Educ. Psychol. Rev. 34, 1919–1954. doi: 10.1007/s10648-022-09702-4

[ref103] Wilde J., (1996). Rational Counseling with School-Aged Populations: A Practical Guide, Bristol: Accelerated Development.

[ref104] Willower D. J. Eidell T. L. Hoy W. K. (1967). The School and Pupil Control Ideology. Penn State Studies Monograph. University Park, PA: Pennsylvania State University.

[ref105] Yeo H. J. (2022). The effects of life stress on depression in nursing students: the mediating effect of unconditional self acceptance. J. Korean Soc. Sch. Health. 35, 31–39. doi: 10.15434/kssh.2022.35.1.31

